# Supplementary material for: Acute Chagas disease in Brazil from 2001 to 2018: A nationwide spatiotemporal analysis
Source: PLoS Negl Trop Dis. 2020 Aug 3;14(8):e0008445. doi: 10.1371/journal.pntd.0008445 (PMC7425982; doi:10.1371/journal.pntd.0008445)
Supplement: S1 Checklist — (DOCX) [file pntd.0008445.s001.docx]

**STROBE 2007 (v4) Statement—Checklist of items that should be included in reports of *cross-sectional studies***

“Acute Chagas disease in Brazil from 2001 to 2017: A nationwide spatiotemporal analysis”

| **Section/Topic** | Item # | Recommendation | Reported on page # |
| --- | --- | --- | --- |
| **Title and abstract** | 1 | (*a*) Indicate the study’s design with a commonly used term in the title or the abstract | Page 1, lines 1-2 |
|  |  | (*b*) Provide in the abstract an informative and balanced summary of what was done and what was found | Pages 2-3, lines 32-56 |
| Introduction | | |  |
| Background/rationale | 2 | Explain the scientific background and rationale for the investigation being reported | Pages 6-7, lines 85-111 |
| Objectives | 3 | State specific objectives, including any prespecified hypotheses | Pages 7, lines 112-116 |
| Methods | | |  |
| Study design | 4 | Present key elements of study design early in the paper | Pages 8-9, lines 143-159 |
| Setting | 5 | Describe the setting, locations, and relevant dates, including periods of recruitment, exposure, follow-up, and data collection | Pages 7, lines 121-128  Pages 8-9, lines 143-159 |
| Participants | 6 | (*a*) Give the eligibility criteria, and the sources and methods of selection of participants | Pages 8-9, lines 143-159 |
| Variables | 7 | Clearly define all outcomes, exposures, predictors, potential confounders, and effect modifiers. Give diagnostic criteria, if applicable | Pages 8-9, lines 143-159 |
| Data sources/ measurement | 8* | For each variable of interest, give sources of data and details of methods of assessment (measurement). Describe comparability of assessment methods if there is more than one group | Pages 8-9, lines 143-159  Page 9, lines 162-182 |
| Bias | 9 | Describe any efforts to address potential sources of bias | Page 9, lines 162-182 |
| Study size | 10 | Explain how the study size was arrived at | Page 9, lines 162-182 |
| Quantitative variables | 11 | Explain how quantitative variables were handled in the analyses. If applicable, describe which groupings were chosen and why | Page 9, lines 162-182 |
| Statistical methods | 12 | (*a*) Describe all statistical methods, including those used to control for confounding | Page 9, lines 162-182 |
|  |  | (*b*) Describe any methods used to examine subgroups and interactions | N/A |
|  |  | (*c*) Explain how missing data were addressed | N/A |
|  |  | (*d*) If applicable, describe analytical methods taking account of sampling strategy | Page 9, lines 162-182 |
|  |  | (*e*) Describe any sensitivity analyses | N/A |
| **Results** |  |  |  |
| Participants | 13* | (a) Report numbers of individuals at each stage of study—eg numbers potentially eligible, examined for eligibility, confirmed eligible, included in the study, completing follow-up, and analysed | Page 10, lines 195-205 |
|  |  | (b) Give reasons for non-participation at each stage | N/A |
|  |  | (c) Consider use of a flow diagram | N/A |
| Descriptive data | 14* | (a) Give characteristics of study participants (eg demographic, clinical, social) and information on exposures and potential confounders | Page 10, lines 195-205  Fig 3 |
|  |  | (b) Indicate number of participants with missing data for each variable of interest | Page 10, lines 195-205  Fig 3 |
| Outcome data | 15* | Report numbers of outcome events or summary measures | Page 10, lines 195-205  Fig 3 + Fig 4 |
| Main results | 16 | (*a*) Give unadjusted estimates and, if applicable, confounder-adjusted estimates and their precision (eg, 95% confidence interval). Make clear which confounders were adjusted for and why they were included | Page 10, lines 195-205 |
|  |  | (*b*) Report category boundaries when continuous variables were categorized | N/A |
|  |  | (*c*) If relevant, consider translating estimates of relative risk into absolute risk for a meaningful time period | N/A |
| Other analyses | 17 | Report other analyses done—eg analyses of subgroups and interactions, and sensitivity analyses | Fig 3 |
| Discussion |  |  |  |
| Key results | 18 | Summarise key results with reference to study objectives | Pages 13-14, lines 227-297 |
| Limitations | 19 | Discuss limitations of the study, taking into account sources of potential bias or imprecision. Discuss both direction and magnitude of any potential bias | Pages 17-18, lines 369-385 |
| Interpretation | 20 | Give a cautious overall interpretation of results considering objectives, limitations, multiplicity of analyses, results from similar studies, and other relevant evidence | Pages 13-18, lines 277-398 |
| Generalisability | 21 | Discuss the generalisability (external validity) of the study results | Page 18, lines 383-385 |
| Other information |  |  |  |
| Funding | 22 | Give the source of funding and the role of the funders for the present study and, if applicable, for the original study on which the present article is based | In submission information |

*Give information separately for cases and controls in case-control studies and, if applicable, for exposed and unexposed groups in cohort and cross-sectional studies.

**Note:** An Explanation and Elaboration article discusses each checklist item and gives methodological background and published examples of transparent reporting. The STROBE checklist is best used in conjunction with this article (freely available on the Web sites of PLoS Medicine at http://www.plosmedicine.org/, Annals of Internal Medicine at http://www.annals.org/, and Epidemiology at http://www.epidem.com/). Information on the STROBE Initiative is available at www.strobe-statement.org.
