# Supplementary material for: Acute Chagas disease in Brazil from 2001 to 2018: A nationwide spatiotemporal analysis
Source: PLoS Negl Trop Dis. 2020 Aug 3;14(8):e0008445. doi: 10.1371/journal.pntd.0008445 (PMC7425982; doi:10.1371/journal.pntd.0008445)
Supplement: Attachment — Submitted filename: Response.pdf [file pntd.0008445.s005.pdf]

Dear Editor,

Thank you very much for your attention and extension of the time to send the final version of the manuscript.

The manuscript was updated according to the update done by the Brazilian Ministry of Health on the SINAN database. Additionally, the reply to reviewers is also below.

Update:

Title:

Before: "Acute Chagas disease in Brazil from 2001 to 2017: A nationwide spatiotemporal analysis"

After (line 1-2): "Acute Chagas disease in Brazil from 2001 to 2018: A nationwide spatiotemporal analysis"

Abstract:

Before: "…In this nationwide population-based ecological all cases of ACD reported in Brazil between 2001 and 2017 were included. Epidemiological characteristics and time trends were analyzed through joinpoint regression models and spatial distribution using microregions as the unit of analysis. A total of 4,250 cases of ACD were recorded during the period under study. The annual incidence rate in Brazil was 1.58 per 100,000 inhabitants/year. Three statistically significant changes in time trends were identified: a rapid increase prior to 2004 (Period 1), a stable drop from 2004 to 2013 (Period 2), followed by another increasing trend after 2013 (Period 3)…"

After (line 37-44): "…In this nationwide population-based ecological all cases of ACD reported in Brazil between 2001 and 2018 were included. Epidemiological characteristics and time trends were analyzed through joinpoint regression models and spatial distribution using microregions as the unit of analysis. A total of 5,184 cases of ACD were recorded during the period under study. The annual incidence rate in Brazil was 0.16 per 100,000 inhabitants/year. Three statistically significant changes in time trends were identified: a rapid increase prior to 2005 (Period 1), a stable drop from 2005 to 2009 (Period 2), followed by another increasing trend after 2009 (Period 3)…"

Author Summary:

Before: "…and evaluated relevant epidemiological indicators throughout Brazil from 2001 to 2017. The data present here may contribute to surveillance actions designed at preventing new CD cases. We observed 4,250 cases of ACD during the period under study. The annual incidence rate in Brazil was 1.58 per 100,000 inhabitants/year. Three distinct epidemiological periods were identified: a rapid increase prior to 2004 (Period 1), a stable drop from 2004 to 2013 (Period 2), followed by another increasing trend after 2013 (Period 3)…"

After (line 70-76): "… and evaluated relevant epidemiological indicators throughout Brazil from 2001 to 2018. The data present here may contribute to surveillance actions designed at preventing new CD cases. We observed 5,184 cases of ACD during the period under study. The annual incidence rate in Brazil was 0.16 per 100,000 inhabitants/year. Three distinct epidemiological periods were identified: a rapid increase prior to 2005 (Period 1), a stable drop from 2005 to 2009 (Period 2), followed by another increasing trend after 2009 (Period 3)…"

Introduction:

Before: "Considering that epidemiological data related to CD is publicly available, analysis can aid in the prioritization of regional epidemiological disease surveillance efforts. With the aim of contributing to surveillance actions designed at preventing new CD cases, our results detail the spatiotemporal distribution of notified cases of ACD and evaluate relevant epidemiological indicators throughout Brazil from 2001 to 2017."

After (line 111-115): "Considering that epidemiological data related to CD is publicly available, analysis can aid in the prioritization of regional epidemiological disease surveillance efforts. With the aim of contributing to surveillance actions designed at preventing new CD cases, our results detail the spatiotemporal distribution of notified cases of ACD and evaluate relevant epidemiological indicators throughout Brazil from 2001 to 2018."

Material and Methods / Study population and design

Before: "…Chagas disease surveillance encompasses the compulsory notification to SINAN of all confirmed cases of ACD; all cases reported between 2001 and 2017 in the 558 Brazilian microregions were included…"

After (line 144-146): "…Chagas disease surveillance encompasses the compulsory notification to SINAN of all confirmed cases of ACD; all cases reported between 2001 and 2018 in the 558 Brazilian microregions were included…"

Material and Methods / Data analysis

Before: "…Three-year moving averages were calculated between 2001 and 2017 [15]…"

After (line 165-166): "…Three-year moving averages were calculated between 2001 and 2018 [15].…"

Results / Spatial distribution of acute CD

Before: "Between 2001 and 2017, 4,250 cases of ACD were reported to SINAN. At least one case of ACD was reported in 300 of 558 (53.8%) microregions, corresponding to 666 municipalities and a total population size of 126,211,631 inhabitants (60.8% of the Brazilian population). The annual rate of reported cases in Brazil between 2001 and 2017 was 0.16 per 100,000 inhabitants/year, ranging from 0.06 to 0.30 notified cases per 100,000 inhabitants. Joinpoint regression analysis from 2001 to 2017 revealed three statistically significant changes in tendency: a rapid increase in cases from 2002 to 2004,

a considerable drop from 2004 to 2013, followed by another increase during the period from 2013 to 2016 (Figure 2)…"

After (line 194-202): "Between 2001 and 2018, 5,184 cases of ACD were reported to SINAN. At least one case of ACD was reported in 307 of 558 (55.0%) microregions, corresponding to 3,238 municipalities and a total population size of 158,363,480 inhabitants (76.3% of the Brazilian population). The annual rate of reported cases in Brazil between 2001 and 2018 was 0.16 per 100,000 inhabitants/year, ranging from 0.07 to 0.32 notified cases per 100,000 inhabitants. Joinpoint regression analysis from 2001 to 2018 revealed three statistically significant changes in tendency: a rapid increase in cases from 2001 to 2005, a considerable drop from 2005 to 2009, followed by another increase during the period from 2009 to 2018 (Figure 2)…"

Figure 2

Before:

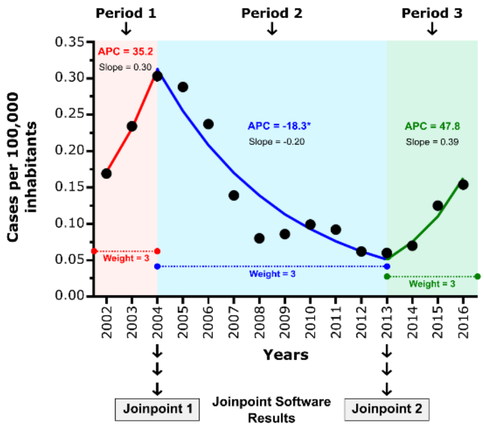

After:

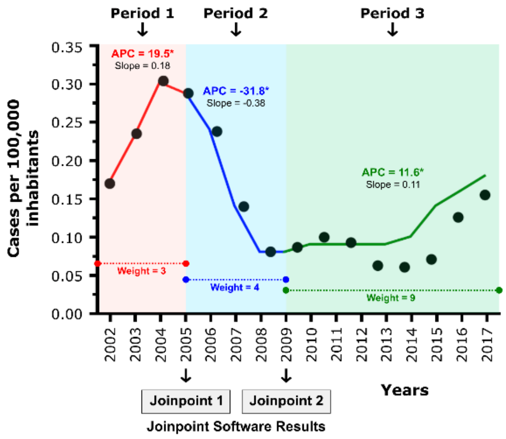

Legend - Figure 2

Before: "**Fig 2. Global rates (expressed at 3-year moving averages) of ACD notifications in Brazil per 100,000 inhabitants (2001 to 2017) according to SINAN.** Three periods were identified by Joinpoint regression using APC (Annual Percentage Change) calculations: Period 1 (2002-2004), Period 2 (2004-2013) and Period 3 (2013-2016)."

After (line 194-202):

"**Fig 2. Global rates (expressed at 3-year moving averages) of ACD notifications in Brazil per 100,000 inhabitants (2001 to 2018) according to SINAN.** Three periods were identified by Joinpoint regression using APC (Annual Percentage Change) calculations: Period 1 (2001-2005), Period 2 (2005-2009) and Period 3 (2009-2018)." ---

----

Results / Spatial distribution of acute CD

Before: "…In the North macroregion, ACD was generally reported more often in males than females…"

After (line 228-229): "…In the North macroregion, ACD was similarly reported in males and females…"

Results / Spatial distribution of acute CD

Before: "…Vectorial transmission was the main route reported during Period 1, while oral transmission increased significantly in the North as a result of many case notifications originating from the states of Pará (Furos de Breves - 109 cases; Belém - 262 cases; Cametá -197 cases) and Amapá (Macapá - 85 cases) in Period 2, as well as in Pará (Portel - 41 cases; Furo de Breves - 175 cases; Belém - 265 cases; Cametá - 154) in Period 3…"

After (line 239-243): "…Vectorial transmission was the main route reported during Period 1, while oral transmission increased significantly in the North as a result of many case notifications originating from the states of Pará (Furos de Breves - 80 cases; Belém - 232 cases; Cametá - 88 cases) and Amapá (Macapá - 93 cases) in Period 2, as well as in Pará (Portel - 63 cases; Furo de Breves - 359 cases; Belém - 848 cases; Cametá - 500 cases) in Period 3…"

Figure 3

Before:

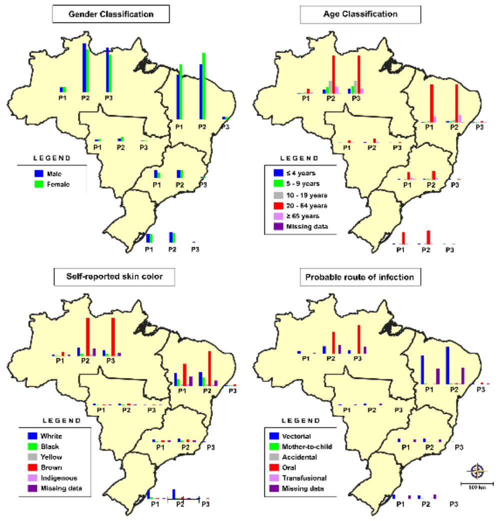

After:

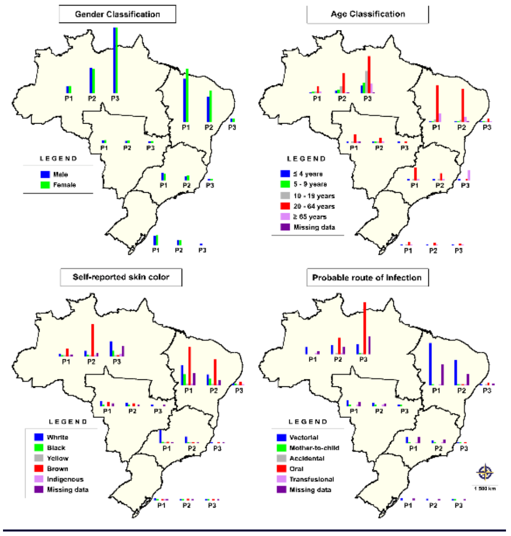

Results / Spatial distribution of acute CD

Before: "The spatiotemporal distribution of reported rates of ACD infection is shown at 15 distinct time points in Figure 4. Overall, significant changes in the incidence of CD throughout Brazil are evidenced over time. The number of positive cases was found to increase in the state of Pará (North macroregion), especially after 2007. Indeed, in 2016, high numbers of cases were reported in the microregions of Furos de Breves (169.01 cases per 100,000 inhabitants), Cametá (82.52 cases per 100,000 inhabitants), Portel (61.79 cases per 100,000 inhabitants) and Belém (27.76 cases per 100,000 inhabitants). …"

After (line 257-263): "The spatiotemporal distribution of reported rates of ACD infection is shown at 16 distinct time points in Figure 4. Overall, significant changes in the incidence of CD throughout Brazil are evidenced over time. The number of positive cases

was found to increase in the state of Pará (North macroregion), especially after 2007. Indeed, in 2016, high numbers of cases were reported in the microregions of Furos de Breves (170.78 cases per 100,000 inhabitants), Cametá (80.86 cases per 100,000 inhabitants), Portel (63.14 cases per 100,000 inhabitants) and Belém (28.16 cases per 100,000 inhabitants)…"

Results / Spatial distribution of acute CD

Before: "…Barreiras (102.64 in 2004), Santa Maria da Vitória (48.98 in 2005) and Cotegipe (42.8 in 2005). Similarly, Itaberaba (Bahia), a microregion located in Chapada Diamantina, a semi-arid area in the state's central region, presented 45.64 cases per 100,000 inhabitants in 2004. Other microregions with a similar climate located in the Brazilian Northeast, e.g. Pau dos Ferros and Médio Oeste (Rio Grande do Norte) reported 73.51 and 111.55 cases per 100,000 inhabitants in 2005…"

After (line 272-278): "…Barreiras (102.64 in 2004), Santa Maria da Vitória (48.08 in 2005) and Cotegipe (42.8 in 2005). Similarly, Itaberaba (Bahia), a microregion located in Chapada Diamantina, a semi-arid area in the state's central region, presented 45.64 cases per 100,000 inhabitants in 2004. Other microregions with a similar climate located in the Brazilian Northeast, e.g. Pau dos Ferros and Médio Oeste (Rio Grande do Norte) reported 73.51 and 112.36 cases per 100,000 inhabitants in 2005…"

Figure 4

Before:

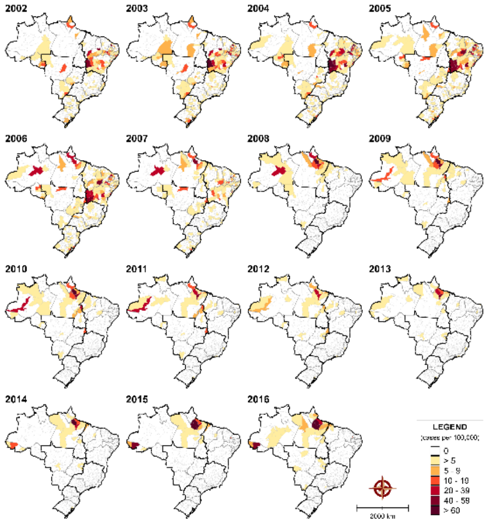

After:

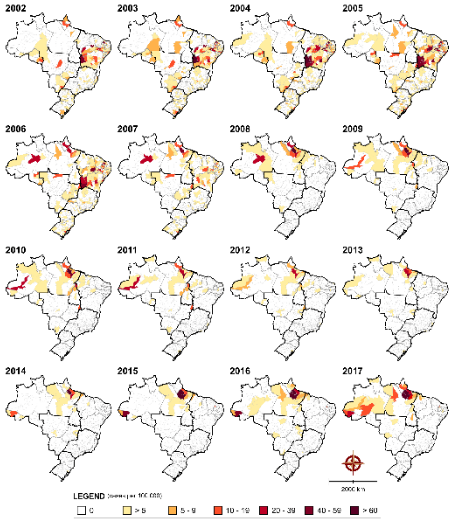

Discussion

Before: "…Despite the statistical similarities observed between genders [19], our findings revealed that more men were infected in the North of Brazil, whereas more women were infected in the Northeast…"

After (line 313-315): "…Despite the statistical similarities observed between genders [19], our findings revealed that both women and men were infected in the North of Brazil, whereas more women were infected in the Northeast.…"

Discussion

Before: "…Despite these drawbacks, we nonetheless consider the results presented herein to be of high validity and highly representative, since all cases of acute CD reported during the period from 2001 to 2017 were included in Brazil, a country of substantial size…"

After (line 401-404): "…Despite these drawbacks, we nonetheless consider the results presented herein to be of high validity and highly representative, since all cases of acute CD reported during the period from 2001 to 2018 were included in Brazil, a country of substantial size.…."

Reply to the considerations of Reviewers:

Reviewer #2: Table S2: These are the numbers as used for the Figure. Please include a standard table in the manuscript presenting data of all cases, without stratification by region and period! Include relative frequencies! Variables: age, gender, skin color, route of infection, region, year. Reviewer #2: In general, the manuscript is acceptable. However, inclusion of the table with description of the cases, as described above, is crucial.

Reply: We thank the reviewer for his/her observation. We have inserted a table (Table 1) including the description of all ACD reported cases. We have considered sociodemographic variables (gender and age classification), self-reported skin color, probable route of infection, year of notification, and Brazilian regions of notification. We have also included the relative frequency for each variable. A new statement was included, as transcript below:

(Line 221-225): Sociodemographic variables, self-reported skin color and probable route of *T. cruzi* infection of all ACD notifications are summarized in Table 1. Of the 5,184 cases of ACD, 2,607 (50.29%) were male and 2,575 (49.67%) female. Most cases (70.68%) were middle-aged adults from 20 to 64 years. However, infected cases were also found in all ages. In the total sample, self-reported skin color was presented in the following proportions of cases: brown/mixed-race (60.59%), white (20.71%), black (7.25%), indigenous (0.98) and yellow (0.79%). According to the probable route of infection, overall, vectorial (35.39%) and oral (38.27%) were the most important. It is noteworthy that the North region is responsible for 55.62% of cases in Brazil.

Table 1. Data stratified by sociodemographic variables, self-reported skin color and probable route of *Trypanosoma cruzi* infection during the period under study (2001-2018) of acute Chagas disease notifications*.

Category	Division	Frequency	Percentage (%)
Gender classification	Male	2,607	50.29

	Female	2,575	49.67
	No data	2	0.04
Age classification	≤ 4	213	4.11
	5 - 9	256	4.94
	10 - 19	572	11.03
	20 - 64	3,664	70.68
	≥ 65	475	9.16
	No data	4	0.08
Self-reported skin color	White	1,073	20.71
	Black	376	7.25
	Yellow	41	0.79
	Brown	3,141	60.59
	Indigenous	51	0.98
	No data	502	9.68
Probable route of infection	Vectorial	1,834	35.39
	Mother-to-child	19	0.37
	Accidental	4	0.08
	Oral	1,984	38.27
	Transfusional	16	0.31
	No data	1,327	25.60
Year of notification	2001	59	1.14
	2002	246	4.75
	2003	579	11.17
	2004	419	8.08
	2005	629	12.13
	2006	544	10.48
	2007	156	3.01
	2008	104	2.01
	2009	220	4.24
	2010	130	2.51
	2011	190	3.67
	2012	189	3.65
	2013	163	3.14
	2014	196	3.78
	2015	268	5.17
	2016	372	7.18
	2017	340	6.56
	2018	380	7.33
Brazilian regions	Central-West	112	2.16
	North	2,883	55.62
	Northeast	1,673	32.27
	South	291	5.61
	Southeast	225	4.34

*Data updated on May 22, 2020.

Sincerely,

Fred Luciano Neves Santos
Corresponding author
