# Supplementary material for: Acute Chagas disease in Brazil from 2001 to 2018: A nationwide spatiotemporal analysis
Source: PLoS Negl Trop Dis. 2020 Aug 3;14(8):e0008445. doi: 10.1371/journal.pntd.0008445 (PMC7425982; doi:10.1371/journal.pntd.0008445)
Supplement: Attachment — Submitted filename: Response.pdf [file pntd.0008445.s006.pdf]

Dear Editors,

Thank you very much for analyzing our manuscript. The replies to Editor's comments are below:

Associate Editor's comments:

I think the authors did a very good job in addressing the major concerns of the reviewers in this new R2 version. However, I have some concerns myself that need to be clarified before acceptance of this manuscript.

Figure 3: I noticed that the revised graph shows datapoints from 2001 to 2017. However, the legend (line 207) states the data are from 2001 to 2018. The same issue applies to Figure 4, where the new maps are from 2002 to 2017, whereas 2018 is missing.

Reply: The data refer to the period from 2001 to 2018, however, the analyzes were performed using a three-year moving average. This strategy minimizes bias in this type of assessment. The three-year average is attributed to the year at the center of the period. For this reason, the years 2001 and 2018 do not appear in figures using the three-year moving average (2018 is not missing). Please see the example below. Explanation for using three-year moving averages available in "Data analysis" subsection (line 165-166): "Three-year moving averages were calculated between 2001 and 2018 [15]".

Three-year moving average	Attributed to the year at the center of the period
2001, 2002, 2003	2002
2002, 2003, 2004	2003
2003, 2004, 2005	2004
2004, 2005, 2006	2005
2005, 2006, 2007	2006
2006, 2007, 2008	2007
2007, 2008, 2009	2008
2008, 2009, 2010	2009
2009, 2010, 2011	2010
2010, 2011, 2012	2011
2011, 2012, 2013	2012
2012, 2013, 2014	2013
2013, 2014, 2015	2014
2014, 2015, 2016	2015
2015, 2016, 2017	2016
2016, 2017, 2018	2017

We completely agree with Editor's comments and we have reviewed the manuscript to incorporate the suggestions.

Figure 3
Before: "Fig 2. Global rates (expressed at 3-year moving averages) of ACD notifications in Brazil per 100,000 inhabitants (2001 to 2018) according to SINAN. Three periods were identified by Joinpoint regression using APC (Annual Percentage Change) calculations: Period 1 (2001-2005), Period 2 (2005-2009) and Period 3 (2009-2018).
After (line 206-209): "Fig 2. Global rates (expressed at 3-year moving averages) of ACD notifications in Brazil per 100,000 inhabitants (2002 to 2017) according to SINAN. Three periods

were identified by Joinpoint regression using APC (Annual Percentage Change) calculations: Period 1 (2001-2005), Period 2 (2005-2009) and Period 3 (2009-2018).

Figure 4

Before: "Fig 4. Spatiotemporal distribution of relative risk of ACD by microregion, based on number of case notifications (SINAN-Brazilian Ministry of Health, Brazil, 2001-2017). Public domain digital maps were obtained from the Brazilian Institute of Geography and Statistics (IBGE) cartographic database in shapefile format (.shp), which was subsequently reformatted and analyzed using QGIS version 3.10 (Geographic Information System, Open Source Geospatial Foundation Project. http://qgis.osgeo.org)."

After (line 282-287): "Fig 4. Spatiotemporal distribution of relative risk of ACD by microregion (expressed at 3-year moving averages), based on number of case notifications (SINAN-Brazilian Ministry of Health, Brazil, 2002-2017). Public domain digital maps were obtained from the Brazilian Institute of Geography and Statistics (IBGE) cartographic database in shapefile format (.shp), which was subsequently reformatted and analyzed using QGIS version 3.10 (Geographic Information System, Open Source Geospatial Foundation Project. http://qgis.osgeo.org)."

On the other hand, Table 1 has data from 2001 to 2018. I understand that some of the official parameters are perhaps not available for 2018 or any other particular year(s), but the manuscript needs to keep a certain consistency throughout it. Please, clarify these discrepancies in the text, just stating when and, if possible, why certain parameter(s) or data for a particular year is/are not available.

Reply: In fact, table 1 has data from 2001 to 2018. This table refers to the absolute number of reported cases stratified according to the parameters previously required by Reviewer 2.
