# Supplementary material for: Acute Chagas disease in Brazil from 2001 to 2018: A nationwide spatiotemporal analysis
Source: PLoS Negl Trop Dis. 2020 Aug 3;14(8):e0008445. doi: 10.1371/journal.pntd.0008445 (PMC7425982; doi:10.1371/journal.pntd.0008445)
Supplement: Attachment — Submitted filename: Response.docx [file pntd.0008445.s004.docx]

**Response to Editor and Reviewers**

Dear Editor,

The response to the questions are below:

“Dear PhD Santos,

Thank you very much for submitting your manuscript "Acute Chagas disease in Brazil from 2001 to 2017: A nationwide spatiotemporal analysis" for consideration at PLOS Neglected Tropical Diseases. As with all papers reviewed by the journal, your manuscript was reviewed by members of the editorial board and by several independent reviewers. In light of the reviews (below this email), we would like to invite the resubmission of a significantly-revised version that takes into account the reviewers' comments.

We cannot make any decision about publication until we have seen the revised manuscript and your response to the reviewers' comments. Your revised manuscript is also likely to be sent to reviewers for further evaluation.

When you are ready to resubmit, please upload the following:

[1] A letter containing a detailed list of your responses to the review comments and a description of the changes you have made in the manuscript. Please note while forming your response, if your article is accepted, you may have the opportunity to make the peer review history publicly available. The record will include editor decision letters (with reviews) and your responses to reviewer comments. If eligible, we will contact you to opt in or out.

[2] Two versions of the revised manuscript: one with either highlights or tracked changes denoting where the text has been changed; the other a clean version (uploaded as the manuscript file).

Important additional instructions are given below your reviewer comments.

Please prepare and submit your revised manuscript within 60 days. If you anticipate any delay, please let us know the expected resubmission date by replying to this email. Please note that revised manuscripts received after the 60-day due date may require evaluation and peer review similar to newly submitted manuscripts.

Thank you again for your submission. We hope that our editorial process has been constructive so far, and we welcome your feedback at any time. Please don't hesitate to contact us if you have any questions or comments.

Sincerely,

Igor C. Almeida

Associate Editor

PLOS Neglected Tropical Diseases

Alain Debrabant

Deputy Editor

PLOS Neglected Tropical Diseases

***********************

**Reviewer's Responses to Questions**

Key Review Criteria Required for Acceptance?

As you describe the new analyses required for acceptance, please consider the following:

**Methods**

-Are the objectives of the study clearly articulated with a clear testable hypothesis stated?

-Is the study design appropriate to address the stated objectives?

-Is the population clearly described and appropriate for the hypothesis being tested?

-Is the sample size sufficient to ensure adequate power to address the hypothesis being tested?

-Were correct statistical analysis used to support conclusions?

-Are there concerns about ethical or regulatory requirements being met?

Reviewer #1: The objectives are clear and in accordance with the retrospective study design. I think the sample size is sufficient is suitable to the tested hypothesis and statistical analysis supports the conclusions. Regarding ethical principles, there are no concerns.

Reviewer #2: Please see general comments below.

--------------------

**Results**

-Does the analysis presented match the analysis plan?

-Are the results clearly and completely presented?

-Are the figures (Tables, Images) of sufficient quality for clarity?

Reviewer #1: The results correspond to the plan and the data are clear and complete. Figures and tables are self-explanatory and quality.

Reviewer #2: Please see general comments below.

--------------------

**Conclusions**

-Are the conclusions supported by the data presented?

-Are the limitations of analysis clearly described?

-Do the authors discuss how these data can be helpful to advance our understanding of the topic under study?

-Is public health relevance addressed?

Reviewer #1: The conclusions are appropriate to the data emphasizing the study limitations due to the quality of the analyzed data and the underreporting of *Trypanosoma cruzi* in the acute phase. Data analysis is well discussed considering epidemiological indicators related to the acute phase of Chagas disease. It also addresses the importance of reporting suspected cases in primary care and public health services, early diagnosis as well as the gaps regarding the availability of the etiological treatment as relevant factors in controlling the transmission of the parasite.

Reviewer #2: Please see general comments below.

--------------------

**Editorial and Data Presentation Modifications?**

Use this section for editorial suggestions as well as relatively minor modifications of existing data that would enhance clarity. If the only modifications needed are minor and/or editorial, you may wish to recommend “Minor Revision” or “Accept”.

Reviewer #1: I suggest reviewing the entire manuscript of the text to make the changes below:

Abstract: Lines 32, 35, 38 41 and 51. Change the acronym CD to ACD so as not to confuse the reader, CD is Chagas disease and ACD is acute Chagas disease.

**Reply:** We have modified the acronyms as requested by the Reviewer.

*Before:*

(Line 32): “In Brazil, acute Chagas disease (CD) surveillance…”

(Line 35): “…patterns of acute CD notifications in…”

(Line 38): “…ecological all cases of acute CD reported in Brazil…”

(Line 41): “…cases of acute CD were recorded during the period…”

(Line 51): “…time cases of acute CD decreased significantly nationwide…”

*After:*

(Line 32): “In Brazil, acute Chagas disease (ACD) surveillance…”

(Line 34): “…patterns of ACD notifications in…”

(Line 38): “…ecological all cases of ACD reported in Brazil…”

(Line 41): “…cases of ACD were recorded during the period…”

(Line 51): “…time cases of ACD decreased significantly nationwide…”

Author summary: Line 63 - Put number 4 in full (numbers 0-10 must be written in full).

**Reply:** We thank the reviewer by his/her observation. We have put the number 4 in full.

*Before:*

(Line 63): “…infection and can last up to 4 months…”

*After:*

(Line 63): “…infection and can last up to four months…”

Lines 69, 70, 73 and 79 - Change the acronym CD to ACD.

**Reply:** We have modified the acronyms as requested by the Reviewer.

*Before:*

(Line 69): “…data related to acute CD is publicly available…”

(Line 70): “…notified cases of acute CD…”

(Line 73): “…cases of acute CD during the period under study…”

(Line 79): …cases of acute CD decreased significantly nationwide…”

*After:*

(Line 69): “…data related to ACD is publicly available…”

(Line 70): “…notified cases of ACD…”

(Line 72): “…cases of ACD during the period under study…”

(Line 78): …cases of ACD decreased significantly nationwide…”

Introduction: Lines 106, 110 and 115 - Change the acronym CD to ACD.

**Reply:** We have modified the acronyms as requested by the Reviewer.

*Before:*

(Line 106): “…cases of acute CD were reported…”

(Line 110): “…and that acute CD was listed as the cause…”

(Line 115): “…notified cases of acute CD and…”

*After:*

(Line 105): “…cases of ACD were reported…”

(Line 109): “…and that ACD was listed as the cause…”

(Line 114): “…notified cases of ACD and…”

Material and Methods: Lines 144, 146, 152, 162 and 176 - Change the acronym CD to ACD.

**Reply:** We have modified the acronyms as requested by the Reviewer.

*Before:*

(Line 144): “…analysis of acute CD notifications aggregated…”

(Line 146): “…confirmed cases of acute CD…”

(Line 152): “…we obtained records for all acute CD notifications…”

(Line 162): “…All acute CD notifications reported…”

(Line 176): “…relative risk of acute CD among the country’s…”

*After:*

(Line 143): “…analysis of ACD notifications aggregated…”

(Line 145): “…confirmed cases of ACD…”

(Line 151): “…we obtained records for all ACD notifications…”

(Line 162): “…All ACD notifications reported…”

(Line 175): “…relative risk of ACD among the country’s…”

Results: Lines 195, 196, 203, 213, 217 and 243 - Change the acronym CD to ACD.

**Reply:** We thank the reviewer by his/her observation. We have modified the acronyms as requested by the Reviewer.

*Before:*

(Line 195): “…4,250 cases of acute CD were reported to SINAN…”

(Line 196): “…acute case of CD was reported in 300 of 558…”

(Line 203): “…Based on these findings, the evaluated acute CD…”

(Line 213): “…In the North macroregion, acute CD was…”

(Line 217): “…of the country, reports of acute CD in the…”

(Line 243): “…reported rates of acute CD infection is shown at…”

*After:*

(Line 194): “…4,250 cases of ACD were reported to SINAN…”

(Line 195): “…case of ACD was reported in 300 of 558…”

(Line 203): “…Based on these findings, the evaluated ACD…”

(Line 212): “…In the North macroregion, ACD was…”

(Line 216): “…of the country, reports of ACD in the…”

(Line 242): “…reported rates of ACD infection is shown at…”

Fig 2 line 207 and Fig 4 line 268 - Do the same.

**Reply:** We have modified the acronyms as requested by the Reviewer.

*Before:*

(Line 207): “…(expressed at 3-year moving averages) of acute CD…”

(Line 268): “…of relative risk of acute CD by microregion…”

*After:*

(Line 206): “…(expressed at 3-year moving averages) of ACD…”

(Line 266): “…of relative risk of ACD by microregion…”

Review legend of Fig. 3 - No map self-reported skin color - Please review Fig 3 - in blue, white is misspelled, rewrite!

**Reply:** We thank the reviewer for his/her observation and we have accordingly modified the legend of Fig. 3 to incorporate the Reviews’ suggestion.

*Before:*

(Line 207): “Fig 3. Analysis of changes in profiles of sociodemographic variables and probable route of *Trypanosoma cruzi* infection by region, stratified according three periods (P1, P2 and P3) of acute Chagas disease notifications as determined by annual percentage changes.”

*After:*

(Line 233-236): “Fig 3. Analysis of changes in profiles of sociodemographic variables, self-reported skin color and probable route of *Trypanosoma cruzi* infection by region, stratified according three periods (P1, P2 and P3) of acute Chagas disease notifications as determined by annual percentage changes.”

Please Review the list of references, space between words, names of authors etc. See manuscript attached.

**Reply:** We have reviewed the list of references. Space between words was removed and the names of authors were double-checked. Unfortunately, we did not access to manuscript attached by the Reviewer.

Reviewer #2: Please see general comments below.

--------------------

**Summary and General Comments**

Use this section to provide overall comments, discuss strengths/weaknesses of the study, novelty, significance, general execution and scholarship. You may also include additional comments for the author, including concerns about dual publication, research ethics, or publication ethics. If requesting major revision, please articulate the new experiments that are needed.

Reviewer #1: This study addresses epidemiological data related to acute Chagas disease that are publicly available, analyzed the spatiotemporal distribution of notified cases of acute Chagas disease and evaluated relevant epidemiological indicators throughout Brazil from 2001 to 2017. Even with a huge difference between the number of confirmed cases of acute Chagas disease and the notification (suspected) cases reported, there was a clear increase in numbers of acute Chagas disease cases during the last decade. The importance of diagnosis and prompt treatment of cases and prolonged follow-up of patients should be strengthened in the increased risk of outbreaks, providing visibility and development of methodologies and well-designed clinical follow-up.

Reviewer #2: This is a sound analysis of acute cases of Chagas Disease during a period of 17 years. Brazil's macro regions were defined as units of analysis. Methods and conclusions are adequate. There are only some comments:

I am missing a general description of all notified cases and request to include a table with all cases diagnosed, according to available variables available such as sex, age, region, transmission route, year/period etc. (Figure 3 only gives a broad information by region/bar charts).

**Reply:** We thank the reviewer for his/her observation. We have added a table (S2 Table) including all diagnosed cases, according to available variables available such as sex, age, region, transmission route, year/period etc.

*Before:*

(Line 211-212): “Figure 3 illustrates changes in the profiles of the sociodemographic variables studied during the three periods identified.”

(Line 579-580): No text.

*After:*

(Line 211-212): “Figure 3 illustrates changes in the profiles of the sociodemographic variables studied during the three periods identified (S1 Table).”

(Line 617-619): “S1 Table: Acute Chagas disease notifications according to sociodemographic variables, self-reported skin color and probable route of Trypanosoma cruzi infection by Brazilian region, stratified according three periods (P1, P2 and P3).”

Acute Chagas Disease often occurs in outbreaks, especially in the case of oral transmission. In addition, a major outbreak may distort geographical analysis. I suggest to include information on major outbreaks that occurred during the study period, in the discussion section of the manuscript, and to discuss the implications adequately.

**Reply:** We concur entirely with the reviewer with respect to the including of further information regarding the outbreaks in Brazil. We have modified the statements to incorporate the reviewer’s suggestion.

*Before:*

(Line 368): “…underreporting of acute CD cases. Indeed, some of the differences…”

*After:*

(Line 368): “…underreporting of acute CD cases and isolated outbreaks. Indeed, some of the differences…”

(Line 381-385): “…which was included beginning in 2006. Outbreaks have occurred in the country during the period of study, which were responsible for isolated ACD cases in Brazil [7,33,36,43–46]. The main impact of outbreaks, especially in cases of oral transmission in the North Brazilian macroregion, refers to distortion of geographical analysis. Despite these drawbacks, we nonetheless consider the results presented herein to

We have also added the following references (lines 585-601):

43.	Bastos CJ, Aras R, Mota G, Reis F, Dias JP, de Jesus RS, et al. Clinical outcomes of thirteen patients with acute Chagas disease acquired through oral transmission from two urban outbreaks in northeastern Brazil. PLoS Negl Trop Dis. 2010;4(6): e711. doi:10.1371/journal.pntd.0000711. PMID: 20559542.

44.	Vargas A, Malta JMAS, Costa VMD, Cláudio LDG, Alves RV, Cordeiro GDS, et al. Investigação de surto de doença de Chagas aguda na região extra-amazônica, Rio Grande do Norte, Brasil, 2016. Cad Saude Publica. 2018; 34(1): e00006517. doi:10.1590/0102-311X00006517. PMID: 29412311. Portuguese.

45.	Steindel M, Kramer Pacheco L, Scholl D, Soares M, de Moraes MH, Eger I, et al. Characterization of *Trypanosoma cruzi* isolated from humans, vectors, and animal reservoirs following an outbreak of acute human Chagas disease in Santa Catarina State, Brazil. Diagn Microbiol Infect Dis. 2008; 60(1): 25-32. doi:10.1016/j.diagmicrobio.2007.07.016. PMID: 17889480.

46.	Dias JP, Bastos C, Araújo E, Mascarenhas AV, Netto E, Grassi F, et al. Acute Chagas disease outbreak associated with oral transmission. Rev Soc Bras Med Trop. 2008; 41(3): 296-300. doi: 10.1590/s0037-86822008000300014. PMID: 18719812.

This manuscript is written by 15 authors, which is rather unusual for a study analyzing easily available secondary data. However, a description of author contributions is missing. Please include.

**Reply:** We thank the reviewer by his/her observation. A description of author contributions was already available in the Plos Neglected submission system. Our study started in 2016 however we had to stop the analysis at different times. Some students who started the analyzes in 2016 left our laboratory and others joined our team. For this reason, the number of authors is slightly larger than usual. A list with the description of each author contributions is replicated bellow:

**Conceptualization:** Yara M. Gomes, Fred L.N. Santos

**Data curation:** Fred L.N. Santos

**Formal analysis:** Emily F. Santos, Ângelo A.O. Silva, Leonardo M. Leony, Natália E.M. Freitas, Rodrigo P. Del-Rei, Wayner V. Souza, Alejandro L. Ostermayer, Veruska M. Costa, Alberto N. Ramos Jr, Andrea S. Souza, Fred L.N. Santos

**Funding acquisition:** Fred L.N. Santos

**Investigation:** Emily F. Santos, Ramona T. Daltro, Yara M. Gomes, Fred L.N. Santos

**Methodology:** Emily F. Santos, Leonardo M. Leony, Natália E.M. Freitas, Ramona T. Daltro, Fred L.N. Santos

**Project administration:** Fred L.N. Santos

**Resources:** Fred L.N. Santos

**Supervision:** Fred L.N. Santos

**Validation:** Emily F. Santos, Fred L.N. Santos

**Visualization:** Emily F. Santos, Ângelo A.O. Silva, Fred L.N. Santos

**Writing – original draft:** Emily F. Santos, Ângelo A.O. Silva, Leonardo M. Leony, Natália E.M. Freitas, Ramona T. Daltro, Carlos G. Regis-Silva, Rodrigo P. Del-Rei, Wayner V. Souza, Alejandro L. Ostermayer, Veruska M. Costa, Rafaella A. Silva, Alberto N. Ramos Jr, Yara M. Gomes, Fred L.N. Santos

**Writing – review & editing:** Wayner V. Souza, Veruska M. Costa, Alberto N. Ramos Jr, Andrea S. Souza, Yara M. Gomes, Fred L.N. Santos

Minor comments:

Lines 85/86: Correct ... “by the Kinetoplastid hemoflagellated *Trypanosoma cruzi*.” Into “by the hemoflagellated kinetoplastid *Trypanosoma cruzi*”.

**Reply:** We have modified the text as requested by the Reviewer.

*Before:*

(Line 85-86): “…by the Kinetoplastid hemoflagellated *Trypanosoma cruzi*…”

*After:*

(Line 85-86): “…by the hemoflagellated kinetoplastid *Trypanosoma cruzi*…”

Line 301: should be “previous study” – please correct

**Reply:** We have modified the text as requested by the Reviewer.

*Before:*

(Line 301): “…A previously study identified …”

*After:*

(Line 299): “…A previous study identified …”

--------------------

PLOS authors have the option to publish the peer review history of their article (what does this mean?). If published, this will include your full peer review and any attached files.

If you choose “no”, your identity will remain anonymous but your review may still be made public.

Do you want your identity to be public for this peer review? For information about this choice, including consent withdrawal, please see our Privacy Policy.

Reviewer #1: No

Reviewer #2: No

Figure Files:

While revising your submission, please upload your figure files to the Preflight Analysis and Conversion Engine (PACE) digital diagnostic tool, https://pacev2.apexcovantage.com. PACE helps ensure that figures meet PLOS requirements. To use PACE, you must first register as a user. Then, login and navigate to the UPLOAD tab, where you will find detailed instructions on how to use the tool. If you encounter any issues or have any questions when using PACE, please email us at figures@plos.org.

Data Requirements:

Please note that, as a condition of publication, PLOS' data policy requires that you make available all data used to draw the conclusions outlined in your manuscript. Data must be deposited in an appropriate repository, included within the body of the manuscript, or uploaded as supporting information. This includes all numerical values that were used to generate graphs, histograms etc.. For an example see here: http://www.plosbiology.org/article/info%3Adoi%2F10.1371%2Fjournal.pbio.1001908#s5.

Reproducibility:

To enhance the reproducibility of your results, PLOS recommends that you deposit laboratory protocols in protocols.io, where a protocol can be assigned its own identifier (DOI) such that it can be cited independently in the future. For instructions see https://journals.plos.org/plosntds/s/submission-guidelines#loc-methods

__________________________________________________

In compliance with data protection regulations, you may request that we remove your personal registration details at any time. (Use the following URL: https://www.editorialmanager.com/pntd/login.asp?a=r). Please contact the publication office if you have any questions.

*********************************************************************
